# Low-Grade Endemicity of Opisthorchiasis, Yangon, Myanmar

**DOI:** 10.3201/eid2507.190495

**Published:** 2019-07

**Authors:** Woon-Mok Sohn, Bong-Kwang Jung, Sung-Jong Hong, Keon-Hoon Lee, Jong-Bok Park, Hyun-Seung Kim, Seon Cho, Thi Thi Htoon, Htay Htay Tin, Jong-Yil Chai

**Affiliations:** Gyeongsang National University College of Medicine, Jinju, South Korea (W.-M. Sohn);; Korea Association of Health Promotion, Seoul, South Korea (B.-K. Jung, K.-H. Lee, J.-B. Park, H.-S. Kim, S. Cho, J.-Y. Chai); Chung-Ang University, Seoul (S.-J. Hong);; National Health Laboratory, Yangon, Myanmar (T.T. Htoon, H.H. Tin)

**Keywords:** Opisthorchis viverrini, cholangiocarcinoma, liver fluke, adult worm recovery, Metacercaria, Myanmar, parasites, helminths, zoonoses

## Abstract

We performed an epidemiologic survey of opisthorchiasis in Yangon, Myanmar. The fecal egg-positive rate of residents was 0.7%, and we recovered an adult fluke after chemotherapy and purging of an egg-positive resident. We detected *Opisthorchis viverrini* metacercariae in freshwater fish. We found the Yangon area to have low-grade endemicity of opisthorchiasis.

The liver fluke *Opisthorchis viverrini*, a well-known cause of cholangiocarcinoma, is distributed predominantly in Southeast Asia countries (*1**,**2*). In Myanmar, health officials thought that opisthorchiasis might not occur because the population traditionally does not consume raw or undercooked fish. However, 2 recent reports have documented the presence of *O. viverrini* eggs or flukes in Myanmar (*3**,**4*). In 2017, a molecular study detected a mitochondrial cytochrome *c* oxidase subunit I (*cox1*) gene of *O. viverrini* from the fecal samples of persons in a rural area near Yangon (*3*); however, adult flukes were not recovered from the egg-positive persons. Another study in 2018 detected *O. viverrini* metacercariae from freshwater fish (*Puntius brevis*) caught in central Myanmar and obtained adult flukes from experimentally infected hamsters (*4*).

We recently observed a low-grade endemicity of *O. viverrini* infection among residents in the Yangon area. We also recovered an adult fluke (Appendix Figure, panel A) from an egg-positive resident and detected metacercariae in freshwater fish caught in Yangon.

In December 2015, we performed fecal examinations on 2,057 residents in 3 districts of Yangon (North Dagon, South Dagon, and Hlaing-Thayar) by using the Kato–Katz technique. The total number of helminth egg–positive cases was 484 (23.5%); we recovered eggs of *Trichuris trichiura* whipworms (13.3%), *Ascaris lumbricoides* roundworms (8.1%), *Enterobius vermicularis* pinworms (0.9%), *O. viverrini* flukes (0.7%), and other helminth species (0.5%) (Table).

**Table:** Rates of helminth egg infection, by species, among 2,057 persons in 3 districts of Yangon, Myanmar

District	No. persons examined	No. (%) positive					
		*Ascaris lumbricoides*	*Trichuris trichiura*	*Enterobius vermicularis*	*Opisthorchis viverrini*	Other*	Total
Hlaing-Thayar	682	17 (2.5)	90 (13.2)	2 (0.3)	2 (0.3)	2 (0.3)	113 (16.6)
South Dagon	672	83 (13.2)	90 (14.4)	11 (1.8)	8 (1.3)	4 (0.6)	196 (31.3)
North Dagon	748	66 (8.8)	94 (12.6)	6 (0.8)	4 (0.5)	5 (0.7)	175 (23.4)
Total	2,057	166 (8.1)	274 (13.3)	19 (0.9)	14 (0.7)	11 (0.5)	484 (23.5)

*Includes 2 cases of hookworm infection and 1 case each of *Taenia* sp. and *Trichostrongylus* sp. infection.

Among the 14 residents positive for *O. viverrini* eggs (some possibly having mixed infections with minute intestinal fluke species such as *Haplorchis* spp.) (Table; Appendix Figure, panel B), 2 agreed to undergo worm recovery after treatment with praziquantel (40 mg/kg in a single dose) and purging with 25–30 g of MgSO_4_. Fecal examination and anthelmintic treatment of the residents were officially approved by Myanmar’s Ministry of Health and Sport, under the agreement of the South Korea–Myanmar International Collaboration on Intestinal Parasite Control for Schoolchildren in Myanmar (Ethics Review Committee approval no. 005117). Informed consent was received from each person.

The procedure of the worm recovery was as described previously (*5*). One adult fluke that looked like a liver fluke was recovered from 1 of these 2 residents. Minute intestinal fluke species, including *Haplorchis* spp., were not recovered. The adult fluke (Appendix Figure, panel A) was slender (11.1 × 1.5 mm) and had a small oral sucker (0.20 × 0.29 mm), large ventral sucker (0.51 × 0.59 mm), lobed ovary, 2 lobed testes, and a well-developed uterus with numerous eggs (25 × 14 μm). We confirmed the fluke to be an adult specimen of *O. viverrini*.

We also examined 10 species of freshwater fish (n = 160) purchased in a local market of North Dagon to detect *O. viverrini* metacercariae. The fish were transported on ice to Gyeongsang National University College of Medicine (Jinju, South Korea), and examined by using the artificial digestion method (*6*). We detected *O. viverrini* metacercariae in 4 species of fish (forest snakehead [*Channa lucius*], 5/5, 100%; striped snakehead [*C. striata*] 1/29, 3.5%; climbing perch [*Anabas testudineus*] 1/14, 7.1%; and unspecified *Puntioplites* sp., 1/15, 6.7%) (Appendix Figure, panels C, D). In forest snakehead fish, the average metacercarial density per fish was 24.4 (range 1–52). The metacercariae were round to elliptical and were 150–188 μm (average 165 μm) × 98–140 μm (average 122 μm) in size.

The metacercariae were fed orally to 2 golden hamsters (*Mesocricetus auratus*) to recover adult flukes. At day 50 postinfection, 20 adult flukes were recovered from the biliary tracts of the hamsters. The animal experiment was performed in accordance with the guidelines of Gyeongsang National University College of Medicine. The adult flukes were slender (average size 5.1 × 1.2 mm) and had the characteristic features of *O. viverrini* (Appendix Figure, panel F).

Opisthorchiasis is one of the most prevalent foodborne helminthiases in Thailand, Laos, Cambodia, and Vietnam (*2**,**5**–**9*). For example, in Laos, opisthorchiasis is prevalent in the central and southern lowlands along the Mekong River, including Vientiane Municipality and Savannakhet Province, where the rates of *O. viverrini* egg recovery (mixed with some minute intestinal flukes) among residents along rivers were 53.3% (Vientiane) and 67.1% (Savannakhet) (*5**,**7*). In Cambodia, eastern localities (e.g., Kratie Province, 4.6% egg-positive rate) and southern localities (Kampong Cham Province, 24.0% egg-positive rate, and Takeo Province, 23.8%–47.5% egg-positive rates) along the Mekong River were found to be endemic foci (*8**,**9*). From 2 egg-positive residents in Takeo Province, 34 adult *O. viverrini* flukeswere recovered (*10*).

In our study, the *O. viverrini* egg-positive rate of residents in surveyed areas of Myanmar was 0.7%, much lower than the 4.6%–67.1% rates in Laos and Cambodia (*5**–**9*). Also, only 1 adult fluke was recovered in 1 egg-positive case, whereas 34 adult specimens were recovered in 2 residents in Cambodia (*10*). Thus, we concluded that the Yangon area of Myanmar has low-grade endemicity of *O. viverrini*.

Appendix Additional information regarding low-grade endemicity of opisthorchiasis, Yangon, Myanmar.
