# Supplementary material for: Low-Grade Endemicity of Opisthorchiasis, Yangon, Myanmar
Source: Emerg Infect Dis. 2019 Jul;25(7):1435–7. doi: 10.3201/eid2507.190495 (PMC6590760; doi:10.3201/eid2507.190495)
Supplement: Appendix — Additional information regarding low-grade endemicity of opisthorchiasis, Yangon, Myanmar. [file 19-0495-Techapp-s1.pdf]

Article DOI: https://doi.org/10.3201/eid2507.190495

# Low-Grade Endemicity of Opisthorchiasis, Yangon, Myanmar

## Appendix

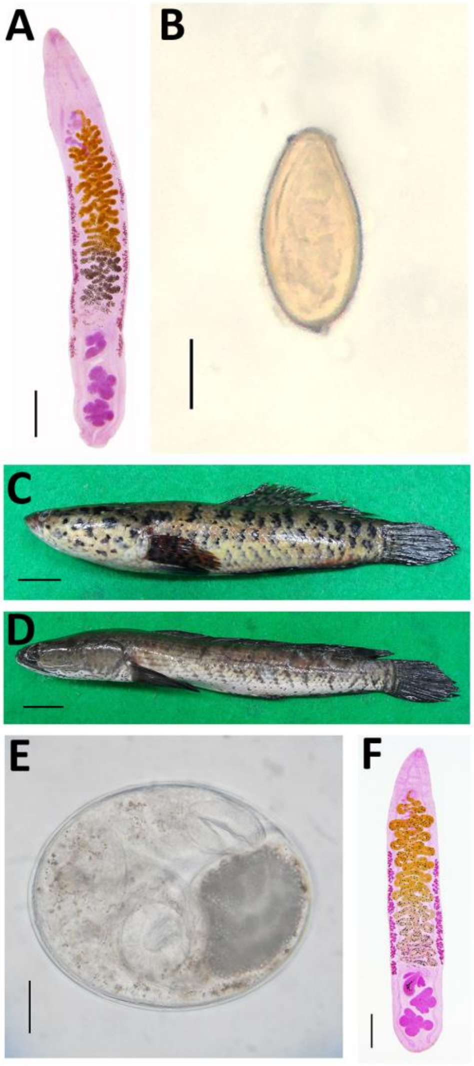


**Appendix Figure.** *Opisthorchis viverrini* from a human case and an experimental hamster, fish hosts, and a metacercaria. A) Adult fluke of *O. viverrini* recovered from a human case (scale bar = 1 mm). B) Egg of *Opisthorchis viverrini* detected by formalin-ether sedimentation of a surveyed resident (scale bar = 10 $\mu$m). C– D) Fish hosts. *Channa lucius* (C) (scale bar = 2 cm); *Channa striata* (D) (scale bar = 2 cm). E) Metacercaria of *O. viverrini* from a fish purchased in Yangon (scale bar = 50 $\mu$m). F) Adult fluke of *O. viverrini* recovered from an experimentally infected hamster (scale bar = 0.5 mm).
